# Common Skin Diseases and Their Psychosocial Impact among Jazan Population, Saudi Arabia: A Cross-Sectional Survey during 2023

**DOI:** 10.3390/medicina59101753

**Published:** 2023-09-30

**Authors:** Mohamed Salih Mahfouz, Ahmad Y. Alqassim, Fanan Adel Hakami, Abrar Khalid Alhazmi, Amjad Mohammed Ashiri, Alaa Marwei Hakami, Linan Mohammed Khormi, Yara Mohammed Adawi, Asmaa Ahmed Jabrah

**Affiliations:** 1 Family and Community Medicine Department, Faculty of Medicine, Jazan University, Jazan 45142, Saudi Arabia; aalqassim@jazanu.edu.sa; 2 Faculty of Medicine, Jazan University, Jazan 45142, Saudi Arabia; fanan14222001@gmail.com (F.A.H.); abrarkhalid_@hotmail.com (A.K.A.); amjad.m.ashiri@gmail.com (A.M.A.); 201908440@stu.jazanu.edu.sa (A.M.H.); 202000744@stu.jazanu.edu.sa (L.M.K.); 202006429@stu.jazanu.edu.sa (Y.M.A.);

**Keywords:** quality of life, skin diseases, psychosocial impact, loneliness, Jazan

## Abstract

*Background and Objectives*: Skin problems are a widespread issue that affects people in both developing and wealthy countries, posing significant public health concerns. These disorders can profoundly impact individuals’ social and psychological well-being. In this study, we aimed to determine the prevalence of the most common skin diseases in the Jazan region of southwestern Saudi Arabia and investigate their impact on patients’ quality of life and psychological and social well-being. *Materials and Methods*: An observational cross-sectional survey conducted among a random sample of 940 adults ≥ 18 years in the Jazan region, Saudi Arabia. Four standardized questionnaires were used for data collection: Patient Health Questionnaire (PHQ-9), a five-dimension questionnaire (EQ-5D), Rosenberg Self-Esteem Scale, and Loneliness Scale (ULS-8). *Results*: The most prevalent skin conditions involved hair loss, which was reported by (61.1%) of the study population. These disorders significantly affected a higher percentage of females (71.9%) compared to males (46.3%) (*p* < 0.001). Melasma was more common in females (14.1%) than in males (4.3%) *p* < 0.001. The lowest prevalence was found for urticaria. Those with acne and hair loss reported higher mean scores on the PHQ-9 Scale than the group without these conditions (*p* < 0.05 for all). Patients with alopecia, acne, and melasma also reported significantly lower self-esteem scores compared to those without these skin conditions (*p* < 0.05 for all). Regarding the activity domain of QoL, participants with skin conditions reported no problems doing usual activities, compared to those without skin problems (*p* < 0.001). *Conclusions*: In conclusion, research revealed that the most prevalent skin condition was hair loss. Further, an association was found between skin diseases and the mental and social well-being of those affected, resulting in a negative impact on their quality of life. The results call for improving the condition of patients with skin diseases, improving their quality of life, and providing appropriate interventions.

## 1. Introduction

Skin diseases are a significant public health issue that affects approximately one-third of the world’s population [1,2]. Skin conditions can drastically reduce a person’s quality of life regarding their psychological health, social interaction, and daily activities. They can also negatively affect their mood, self-worth, and connections with others, which can result in despair and suicidal thoughts. Skin conditions can impact a patient’s daily life, psychological and social well-being, and the lives of their partners, families, friends, and caregivers [3].

Skin diseases have negatively impacted human beings, both in the acceptance of their own image and quality of life [4]. Chronic dermatological disorders, obvious ones that impact appearance, are likely to affect a patient’s self-esteem. Feelings of embarrassment, rejection, anxiety, depression, and social retreat may also result from dermatologic disease’s psychologic effects, visibility, and sociocultural stigma associated with many skin disorders [5].

Skin problems are shared worldwide and constitute a considerable public health burden in developing and wealthy countries. Skin and subcutaneous disorders are the 18th leading cause of Disability Adjusted Life Years (DALYs) globally, accounting for 41.6 million DALYs and 39.0 million Year Lost due to Disability (YLDs) in 2013 [6]. The burden of skin and subcutaneous diseases in the US increased from 821.6 in 1990 to 884.2 in 2017, resulting in a 0.08% change in the age-standardized DALY rate [7]. Between 1990 and 2017, skin and subcutaneous diseases increased by 47% [8]. Acne is believed to affect 9.4% of the world’s population, making it the eighth most common disease worldwide [9]. Alopecia areata is the most common autoimmune disorder, with a lifetime risk of 2% in the global population [10]. The prevalence of psoriasis in adults is estimated by earlier studies to be 0.27% with 95% confidence interval between [0.17 to 0.36] [11]. During the COVID-19 pandemic, individuals with skin diseases faced a unique set of challenges and considerations, including fear of how COVID-19 and vaccination could affect the course of their disease [12,13].

Much research has been conducted during the past two decades which has documented numerous impacts of skin disorders on quality of life, mental health, and social life. Urban et al. (2021) investigated skin disease burden and related socioeconomic conditions in Asia and suggested that some countries with high-income areas have a high prevalence of inflammatory skin diseases while contagious dermatitis was more prevalent in low-income areas [14]. In the United Kingdom, Rutter et al. (2020) in their research on quality of life and psychological impact in people with photodermatoses, revealed that 31–39% of people with photodermatosis have a low quality of life [15]. He et al. (2020) assessed the factors associated with HRQoL in patients with skin diseases in nine hospitals in China and concluded that skin diseases can affect many facets of HRQoL, while the emotional impairment needs more attention [16]. A study in Vietnam by Nguyen et. al. (2018) documented that in terms of HRQoL, pain/discomfort was the most prevalent health concern among individuals with skin conditions, followed by anxiety/depression [17]. A multicenter study focused on congenital ichthyoses conducted in Italy produced a higher overall FDLQI score, which is significantly correlated with a more severe illness score [18].

The profile of skin diseases in Saudi Arabia showed that in terms of general dermatological issues, Saudi Arabia ranks among the most affected countries [19]. A recent meta-analysis conducted by Almohideb (2020) revealed that the mean proportion of pigmentary disorders, extracted from eleven studies, was 16.1%, while the most common pigmentary condition was melanocytic nevi at 54.2%, followed by post-inflammatory pigmentation and vitiligo accounting for 47% and 6%, respectively [20]. Another study that measured quality of life among patients suffering from different dermatological diseases in Saudi Arabia reported that most patients with acne vulgaris, vitiligo, hair disorders, or rosacea reported little or no impact on their quality of life. Nevertheless, urticaria, eczema, and psoriasis were the diseases that reflected the most significant impact on quality of life [21]. Another recent study in KSA documented how those patients with inflammatory skin diseases had significantly worse QoL than those with nevi [22]. A study assessing the prevalence of depression among dermatology clinic patients in a teaching hospital in Saudi Arabia revealed that 10.6% had moderate depressive symptoms, 4.4% had moderate depressive symptoms, and only 0.07 had severe depressive symptoms, while the most common diseases in patients with depressive symptoms were psoriasis and acne [23].

While the psychological aspects of skin diseases are frequently discussed in Western societies, they are infrequently researched in the Arab world. Although some reports have been published on skin conditions and their impact on patients in different regions of KSA, to the best of our knowledge, their impact in Jazan Southwest KSA remains unknown. Hence, we aimed to estimate the prevalence of the most common skin conditions in the Jazan region and assess the association between psychosocial status and the prevalence of self-reported skin diseases in the region. Investigating the profile of these diseases is crucial for disease burden assessment and resource allocation. Further, identifying the factors associated with the increase in the psychological impact of skin diseases is essential for designing intervention programs.

## 2. Materials and Methods

### 2.1. Study Design, Setting, and Period

For this survey, we used a cross-sectional design, as this is an analytical epidemiological study designed to describe the pattern and the psychosocial impact of skin disorders on patients’ quality of life and self-esteem among the Jazan population. This study was conducted in the Jazan region, the second-smallest region of the 13 regions comprising Saudi Arabia. Jazan region is located in the southwest of the country, with a population of over 1.5 million. The region has the highest population density in the Kingdom. The region is sub-divided into 14 governorates. Data were collected between January and February 2023.

### 2.2. Study Population and Sampling Procedures

The target population for this survey included all adults ≥18 years from all Jazan administrative units. Inclusion criteria involved adults living in the Jazan region who agreed to participate in this online survey. The sample size for this research was calculated building on the statistical formula n = [(z^2^ × p × q)]/d^2^. The parameters of the formula included n: initial sample size; p: anticipated population proportion; z: standardized variable that corresponds to 95% confidence level; d: desired marginal error. Using this equation and substituting the parameter’s: prevalence of skin disease of 50% (no available study in the Jazan region), 95% confidence interval, and error not more than 3.5%, the initial sample size was calculated to be 784 participants. The sample size was further increased by 20%, accounting for a non-response rate, which provided a final sample size of 940 participants. To conduct the survey, we used a combination of random and non-random sampling techniques. Firstly, we randomly chose eight out of the fourteen Jazan region administrative units. In the second stage, we used snowball sampling to select participants from each of the selected administrative units. Participants were invited to participate in the study by following the survey link.

### 2.3. Method of Data Collection and Study

A multi-component self-administrated web-based online survey was designed for this research. This study questionnaire was created to measure the psychological impact of skin disorders on the patient’s quality of life and self-estimation. First, background information was collected involving age, gender, place of living, and medical history of skin diseases. All participants were asked about having one of the common skin conditions in Saudi Arabia. In addition, three standardized questionnaires were used to collect information for the study participants.

The PHQ-9 is widely used for its high validity and reliability in diagnostics and measuring the severity of depression [24]. It rates depression based on the self-administered Patient Health Questionnaire. A PHQ-9 score totals 0–4 points, whereby each item of the PHQ-9 is assessed on a 4-point scale (0 = not at all, 1 = several days, 2 = more than half the days, 3 = nearly every day). Scoring between 5–9 points indicates mild depression, 10–14 points indicates moderate depression, 15–19 points indicates moderately severe depression, and 20 or more points indicates severe depression.

The second instrument is the EuroQoL five-dimension questionnaire (EQ-5D), which is one of the most widely used tools to evaluate health-related quality of life (HRQoL). The five dimensions of the EQ-5D are mobility, self-care, usual activities, pain/discomfort, and anxiety/depression. The Arabic version of the EQ-5D was shown to be valid and reliable in an Arabic population with a Cronbach’s α value of 0.72 [25].

The third questionnaire is the Rosenberg Self-Esteem Scale. This 10-item scale measures both positive and negative feelings about the self. The questionnaire is uni-dimensional with responses ranging from strongly agree to strongly disagree (4-point Likert scale format) [26].

The fourth scale is the Arabic version of the short-form University of California Los Angeles Loneliness Scale (ULS-8). This tool is a commonly used tool for measuring loneliness. The Cronbach’s alpha test for the tool provided a level of 0.777 [27].

### 2.4. Measurements and Operational Definitions

The independent variables included demographic, socioeconomic, and clinical characteristics. Demographic and socioeconomic characteristics included gender, age, marital status, educational level, employment status, annual income, and occupation.

The Common skin diseases in this research included dermatitis/eczema, acne vulgaris, alopecia aerate, papulosquamous disorders (psoriasis), pigmentary disorders (vitiligo, chloasma), chronic urticaria, and hair disorders. The list was compiled based on recent studies conducted in Saudi Arabia [19,20,21,22,23,28,29]. For the purpose of this study, the common skin diseases are further divided into two groups; the first group includes chronic skin diseases like vitiligo and eczema, while the second group consists of the remaining diseases mentioned above.

Quality of life (QoL) is defined by the World Health Organization as “an individual’s perception of their position in life in the context of the culture and value systems in which they live and in relation to their goals, expectations, standards, and concerns”.

Depression is a common mental disorder. Globally, it is estimated that 5% of adults suffer from this disorder. It is characterized by persistent sadness and a lack of interest or pleasure in previously rewarding or enjoyable activities. It can also disturb sleep and appetite.

### 2.5. Data Quality Control

To ensure the validity and accuracy of our study, we analyzed scientific literature to determine the appropriate design and methods. We created a questionnaire that aligned with our study objectives and variables, and a trained medical student oversaw the data collection process. We also evaluated the face validity and internal consistency of the data collected and a Cronbach’s α value was produced above 0.70 for all scales.

### 2.6. Statistical Analysis

Data analysis was conducted using the Statistical Package for Social Sciences software, version 25.0 (IBM SPSS Inc., Chicago, IL, USA). Both descriptive and inferential statistics were utilized to address data analysis concerns. Categorical variables were presented as counts and percentages. The prevalence of each skin disease was calculated along with its 95% confidence interval (95% CIs). To determine the association between categorical variables, the chi-squared/Fisher exact tests were performed. Multiple linear regression analysis was carried out to investigate the association of skin diseases with each of the four measured outcomes. The dependent variables in each regression model were EQ-5D-5L and PHQ-9, while the independent variables were the total loneliness score, self-esteem total score, any skin condition, and demographic factors. Results were considered significant for *p*-values less than 0.05.

### 2.7. Ethical Consideration

This study was conducted according to the law of research ethics guidelines of the Helsinki Declaration and the Saudi Bioethics standards guidelines. The ethical approval for this study was obtained from the Jazan University Ethical Committee (Reference # REC-44/06/461). Furthermore, all participants have signed the study consent after being informed about the purpose of the study, and the data will only be used for scientific purposes.

## 3. Results

The estimated response rate for this survey was 92.7% (871 out of 940). Table 1 presents some key characteristics of the participants. The majority of the participants were female (60%) and nearly 40% of them were between the ages of 20–24. In terms of marital status, the majority were single (61%) while (35.7%) were married. Most of the participants had university degrees (70.1%) while only a small percentage had higher degrees (4.4%). In terms of occupation, the majority were unemployed (68.7%) while (24.2%) were employed. The same table also shows the prevalence of two groups of skin diseases: chronic skin diseases and other skin diseases. The prevalence of chronic skin conditions differed significantly according to gender, age group, marital status, educational level, number of children, and presence of chronic conditions (*p* < 0.05 for all). The prevalence of other skin conditions also varied significantly by gender and occupation (*p* < 0.05 for both).

As shown in Table 2, the most prevalent skin diseases were hair loss, which affected (61.1%) of the study population. These disorders affected a higher percentage of females (71.9%) compared to males (46.3%); the difference between males and females was significant at *p* < 0.001. Further, 38.3% of the people being studied had acne. Male participants (39.7%) experienced acne more than female participants (37.5%), but without a statistical difference (*p* > 0.05). Melasma was more common in females (14.1%) than in males (4.3%). Moreover, eczema prevalence was higher in females (16.1%) than in males (14.4%). The prevalence of vitiligo and psoriasis was equal in percentage but more prevalent in males (3.7%) compared to females (2.3%). Males had a higher percentage of alopecia (3.4%) than females (1.3%). Also, males (2%) had a higher prevalence of urticaria than females (0.8%).

Individuals with skin conditions such as eczema, alopecia, acne, melasma, hair loss, and psoriasis have lower average EQ-5D-5L scores compared to those without these conditions (*p* < 0.05 for all). Additionally, those with acne and hair loss reported higher mean scores on the PHQ-9 Scale than the group without these conditions (*p* < 0.05 for all). Patients with alopecia, acne, and melasma also reported significantly lower self-esteem scores compared to those without these skin conditions (*p* < 0.05 for all). Lastly, individuals with skin conditions such as alopecia, acne, melasma, and hair loss reported higher loneliness scale scores compared to those without skin conditions (*p* < 0.05 for all) (Table 3).

Table 4 shows that individuals without skin diseases have a higher level of self-satisfaction compared to those with skin diseases (*p* = 0.002). This study also reveals a statistically significant difference (*p* < 0.001) between those with and without skin problems, indicating the impact of skin diseases on people’s lives. Participants with and without skin diseases have similar levels of responses regarding their ability to do things (*p* = 0.076). However, there is a difference between people with skin diseases and those without when asked if they feel useless sometimes (*p* = 0.001). This study found that 49% of individuals with skin conditions strongly agree to take positive attitudes towards themselves, compared to 63.3% of those without skin conditions (*p* = 0.010). Additionally, 48.1% of those with skin diseases strongly agree to wish they could have more respect for themselves, while 46.7% of those without skin diseases feel the same, with no statistical difference between the two groups (*p* = 0.393).

The comparison of the Health-related Quality (EQ-5D-5L) Scale for participants with and without skin diseases is shown in Table 5. The results indicate that there is no significant difference between the two groups in terms of mobility and self-care (*p* = 0.517 and 0.339, respectively). However, in the activity domain of QoL, 63.2% of participants with skin conditions reported no problems doing usual activities, compared to 82.7% of those without skin problems (*p* < 0.001). In terms of pain, participants with skin conditions reported a 44.2% lower rate of no pain or discomfort compared to those without skin conditions (66.7%), indicating a significant impact on quality of life (*p* < 0.001). Lastly, there was a significant difference in the anxiety domain, as 64.0% of those without skin conditions do not feel anxious compared to only 42.9% of those with skin diseases (*p* < 0.001).

In Table 6, we can see the connection between various factors such as sociodemographic characteristics, quality of life, and mental health. The results of the multiple linear regression analysis showed that several demographics, social factors, and the presence of dermatological skin conditions are significantly associated with EQ-5D-5L and PHQ-9 scores. This study found a positive correlation between age, loneliness total scores, self-esteem total score, and the scores of both scales (*p*-value < 0.001). It is worth noting that the presence of skin diseases is significantly negatively correlated with EQ-5D-5L (*p* < 0.001) and positively correlated with PHQ-9, indicating a positive correlation with gender (*p*-value < 0.001). This shows how skin diseases impact quality of life and mental health. Additionally, this study found a statistically significant positive association between university degrees and both scales (*p* < 0.05).

## 4. Discussion

The main purpose of this study was to estimate the prevalence of the most common skin diseases in the Jazan region and to determine the Psychosocial Impact of Skin Disorders on patients’ quality of life and self-esteem. The highest prevalence of skin conditions in the Jazan region were hair loss which affected 61.1% of the study participants, followed by acne which affected 38.3%.

Hair loss is a common problem that affects both males and females of all ages. It is one of the most common complaints among all patients consulting a dermatologist. The prevalence of hair loss was found to be 61.7% which was comparable to the results of similar studies conducted by K. Shankar et al. in India and P. Tang et al. in Singapore in which the prevalence was found to be 58% and 63%, respectively [30,31].

Our study findings are also consistent with data obtained in a community-based cross-sectional study that was conducted in India in 2018 which found that hair loss was a problem reported by 60.3% of study participants [32]. Another study conducted in Nigeria in 2016 revealed that alopecia 80.6% was more common among people aged 20 to 29 years [33]. In contrast to our findings, a study conducted in 2003 in Assiut Governorate’s rural areas revealed that only 8.4% of the study group had diffuse hair loss [34]. The differences between these rates may be attributed to differences in sample size, study setting, and population characteristics. It is somewhat surprising that females were the most affected 71.9% compared to males 46.3%. This is because women believe that any pattern of hair loss is a disease even if it is a result of a normal phenomenon, but men believe just the opposite.

Acne is estimated to affect 9.4% of the global population, making it the eighth most prevalent disease worldwide [9]. Our data demonstrated that acne was the second most prevalent in our study population at 38.3%. This outcome is comparable to the results of a study conducted by Poli et al., which revealed an acne prevalence of 41% [35]. We also found that the prevalence of eczema among study participants was 15.4% which is to some extent consistent with a study that was conducted in India which showed that eczema was the most common dermatosis accounting for 22% of participants [36].

The results of our study show that skin conditions have an impact on patients’ quality of life particularly in aspects like physical symptoms, feelings, social problems, and self-confidence. Our findings show that the effects of skin conditions on patients’ quality of life varies significantly depending on the patient’s age, with younger adults (aged 20 to 24) experiencing a greater influence than elderly patients. Elderly patients with skin conditions reported higher QoL, perhaps as a result of their propensity to accept and deal with limits more effectively than younger patients. Younger patients, who were more involved in society, would be more worried about how skin lesions seemed to others.

In term of sociodemographic variables, women’s QoL was found to be more impacted than men. Numerous research has demonstrated that women are more frequently affected than males. This result is consistent with two studies from Malaysia and Saudi Arabia [37,38]. However, this finding was in contrast with a previous international study conducted in Columbia that reported no significant differences between the overall QoL scores for both genders [39]. A study also conducted in Al-Qassim province of Saudi Arabia found no significant gender differences in the quality of life of patients with skin diseases [38]. These variations could be the result of diverse demographics, sample techniques, study sites, and the spectrum of diseases studied.

Patients with skin diseases experience a clear reduction in their quality of life, as demonstrated by changes in daily activities and increased anxiety levels, which is consistent with findings from previous studies [15,16,17,21]. Further, individuals with hair loss, acne, and melasma are at a higher risk of experiencing a decline in their quality of life compared to those with other skin conditions, as evidenced by results from many studies [15,16,17,40].

We compared people with skin conditions with those without skin diseases using EQ-5D-5L index scores, PHQ-9 scale, Rosenberg Self-Esteem scale, and UCLA Loneliness scale. The results indicated that acne and melasma have negatively impacted the individuals’ quality of life, psychological health, social interaction, and daily activities compared to those without skin diseases (*p* < 0.05 for all). The explanation for this finding is that acne and melasma are more prevalent among individuals compared to other skin diseases. Similar studies show that participants with skin diseases including acne reported significantly higher PHQ-9 and UCLA Loneliness scale scores and lower LSNS-6 and EQ-5D-5L scores compared to those without any skin diseases [41]. Other studies support our results that depression and anxiety were found to be significantly higher in the patients with apparent skin diseases including acne as compared to the individuals without apparent skin conditions [42,43].

Also, our research revealed that more participants with skin diseases (20.5%) often feel left out, compared to only (13.3%) of those without skin diseases. Also, (31.5%) feel isolated from others among participants with skin disorders compared to (19.3%) of those who did not have skin diseases. The feelings of loneliness among people with skin disorders are reported in previous studies [41,44].

Based on our research, we have found that age, gender, and marital status are the most significant factors associated with the quality of life of individuals with skin diseases. This finding is consistent with the previous studies [17,18,21,22,40].

Our research has shown that skin conditions can have significant social and psychological impacts on individuals. However, health intervention strategies can help patients cope with these challenges. There is increasing evidence to suggest that counseling, psychotherapy, and coping strategies may provide early evidence of improving the quality of life and mental health for patients with skin conditions [45].

### Limitations and Strengths

This study has some limitations that should be considered. Firstly, since it was a cross-sectional study, it was challenging to determine the precise relationship between skin conditions and specific study outcomes, such as mental health and quality of life. Additionally, this study relied on patients self-reporting their condition, rather than a clinical diagnosis, which may have affected the accuracy of the responses. Furthermore, we were unable to establish a correlation between the severity of skin diseases and the evaluated outcomes, as our study did not assess the severity of skin disorders. It is well-documented that skin disease severity is strongly associated with a significant decline in quality of life [16]. Finally, we investigated a group of skin conditions with varying patterns and impacts, so the findings must be understood in this context. Despite these limitations, this research is the first attempt to investigate the impact of skin diseases on patients’ quality of life and their psychological and social well-being. The results of this study may motivate interested researchers to use more robust research designs, such as cohort or interventional studies, which may provide stronger evidence for the psychological impact of skin diseases.

## 5. Conclusions

This study emphasizes the psychosocial impact of skin disorders on patients’ quality of life and provides well-supported scientific data to health authorities. Based on our research, hair loss (61.7%) is the most common skin condition. This study also found associations between skin diseases and the mental and social well-being of those affected, resulting in a negative impact on their quality of life. Skin diseases can affect various areas of a person’s life, leading to increased depression, social isolation, and a reduced quality of life. These findings highlight the need to improve patient care, enhance their quality of life, and provide appropriate interventions. To achieve this, a more comprehensive approach is necessary to minimize the burden of skin disease. Counseling, psychotherapy, and coping strategies may provide early evidence of improving the quality of life and mental health for patients with skin conditions. Further research is needed to understand the factors affecting quality of life, ways to enhance it, and effectively address the comorbidities associated with each skin condition.

## Figures and Tables

**Table 1:** Demographics characteristics and self-report of any skin diseases (n = 871).

Characteristics		All Participants	Chronic Skin Conditions		Other Skin Conditions	
		N (%)	N (%)	*p*-Value	N (%)	*p*-Value
Gender	Male	348 (40.0)	83 (23.9)	**0.014**	227 (65.2)	**<0.001**
	Female	523 (60.0)	165 (31.5)		423 (80.9)	
Age Groups	Less than 18 years	92 (10.6)	19 (20.7)	**<0.001**	67 (72.8)	0.267
	20–24 years	340 (39.0)	79 (23.2)		259 (76.2)	
	25–39 years	300 (34.4)	95 (31.7)		229 (76.3)	
	40 years and more	139 (16.0)	55 (39.6)		95 (68.3)	
Marital Status	Single	531 (61.0)	122 (23.0)	**<0.001**	397 (74.8)	0.961
	Married	311 (35.7)	115 (37.0)		232 (74.6)	
	Divorced/widowed	29 (3.3)	11 (37.9)		21 (72.4)	
Education	Elementary	11 (1.3)	8 (72.7)	**0.009**	8 (72.7)	0.469
	Intermediate/Secondary	211 (24.2)	64 (30.3)		149 (70.6)	
	University degree	611 (70.1)	165 (27.0)		465 (76.1)	
	Postgraduate Degree	38 (4.4)	11 (28.9)		28 (73.7)	
Occupation	Working	273 (31.3)	86 (31.5)	0.181	189 (69.2)	**0.013**
	Not Working	598 (68.7)	162 (27.1)		461 (77.1)	
Number of Children	None	564 (64.8)	129 (22.9)	**<0.001**	426 (75.5)	0.845
	One child	57 (6.5)	17 (29.8)		44 (77.2)	
	Two Children	56 (6.4)	28 (50.0)		40 (71.4)	
	Three Children	70 (8.0)	26 (37.1)		51 (72.9)	
	Four and more	124 (14.2)	48 (38.7)		89 (71.8)	
Family Income	Less than 5000 SR	174 (20.0)	58 (33.3)	0.055	138 (79.3)	0.233
	5000 to 9999 SR	283 (32.5)	81 (28.6)		209 (73.9)	
	10,000 to 14,999 SR	256 (29.4)	77 (30.1)		193 (75.4)	
	20,000 and more SR	158 (18.1)	32 (20.3)		110 (69.6)	
Any chronic Condition	Yes	158 (18.1)	62 (39.2)	<0.001 *	119 (75.3)	0.226
	No	713 (81.9)	186 (26.1)		531 (74.5)	

* chronic skin diseases like vitiligo and eczema, while the second group consists of the remaining diseases mentioned in the methods section.

**Table 2:** Prevalence of skin diseases among study participants by gender (n = 871).

Conditions	Response	Total	Male	Female	*p*-Value
		N%	N%	N%	
Eczema	Yes	134 (15.4)	50 (14.4)	84 (16.1)	0.497
	No	737 (84.6)	298 (85.6)	439 (83.9)	
Alopecia	Yes	19 (2.2)	12 (3.4)	7 (1.3)	**0.037**
	No	852 (97.8)	336 (96.6)	516 (98.7)	
Acne	Yes	334 (38.3)	138 (39.7)	196 (37.5)	0.517
	No	537 (61.7)	210 (60.3)	327 (62.5)	
Melasma	Yes	89 (10.2)	15 (4.3)	74 (14.1)	**<0.001**
	No	782 (89.8)	333 (95.7)	449 (85.9)	
Vitiligo	Yes	25 (2.9)	13 (3.7)	12 (2.3)	0.212
	No	846 (97.1)	335 (96.3)	511 (97.7)	
Urticaria	Yes	11 (1.3)	7 (2.0)	4 (0.8)	0.107
	No	860 (98.7)	341 (98.0)	519 (99.2)	
Hair Loss	Yes	537 (61.7)	161 (46.3)	376 (71.9)	**<0.001**
	No	334 (38.3)	187 (53.7)	147 (28.1)	
Psoriasis	Yes	25 (2.9)	13 (3.7)	12 (2.3)	0.212
	No	846 (97.1)	335 (96.3)	511 (97.7)	

**Table 3:** Comparisons of EQ-5D-5L index scores, PHQ-9, Rosenberg Self-Esteem Scale, and UCLA loneliness scale between participants with and without skin diseases for the study participants (n = 871).

Conditions		EQ-5D-5L Index		PHQ-9 Scale		Self-Esteem Scale		Loneliness Scale	
		Mean (SD)	*p* Value	Mean (SD)	*p* Value	Mean (SD)	*p* Value	Mean (SD)	*p* Value
Eczema	Yes	0.761 (0.24)	**0.003**	9.49 (6.23)	0.110	27.23 (4.73)	0.262	5.64 (1.92)	0.287
	No	0.819 (0.22)		8.58 (5.97)		27.07 (4.82)		5.45 (1.92)	
Alopecia	Yes	0.524 (0.49)	**0.001**	10.00 (6.62)	0.349	23.53 (5.47)	**0.001**	6.26 (2.38)	**0.037**
	No	0.817 (0.21)		8.69 (6.00)		27.17 (4.76)		5.46 (1.91)	
Acne	Yes	0.771 (0.24)	**<0.001**	10.09 (6.12)	**<0.001**	26.21 (4.90)	**<0.001**	5.71 (1.96)	**0.006**
	No	0.835 (0.20)		7.87 (5.79)		27.65 (4.66)		5.34 (1.88)	
Melasma	Yes	0.659 (0.28)	**<0.001**	11.62 (6.17)	**<0.001**	25.78 (5.00)	**0.006**	5.88 (1.83)	**0.020**
	No	0.827 (0.21)		8.39 (5.91)		27.25 (4.76)		5.43 (1.93)	
Vitiligo	Yes	0.788 (0.23)	0.621	9.20 (4.88)	0.687	26.68 (4.63)	0.661	5.16 (1.93)	0.400
	No	0.811 (0.22)		8.71 (6.05)		27.11 (4.81)		5.49 (1.92)	
Urticaria	Yes	0.772 (0.31)	0.572	9.82 (6.29)	0.543	26.18 (6.43)	0.526	5.00 (1.95)	0.406
	No	0.811 (0.22)		8.71 (6.01)		27.11 (4.78)		5.48 (1.92)	
Hair Loss	Yes	0.790 (0.22)	**<0.001**	9.62 (6.01)	**<0.001**	27.06 (4.71)	0.803	5.60 (1.97)	**0.020**
	No	0.842 (0.22)		7.28 (5.75)		27.15 (4.95)		5.29 (1.83)	
Psoriasis	Yes	0.695 (0.35)	**0.009**	9.88 (6.27)	0.329	27.08 (4.80)	0.987	5.24 (1.85)	0.528
	No	0.814 (0.22)		8.69 (6.01)		27.10 (4.80)		5.49 (1.92)	

**Table 4:** Comparisons of Rosenberg Self-Esteem Scale and UCLA Loneliness Scale for participants with and without any skin diseases (n = 871).

	Items		Any Skin Disease		*p*-Value
			No	Yes	
			N (%)	N (%)	
Rosenberg Self-Esteem Scale	On the whole, I am satisfied with myself.	Strongly Agree	82 (54.7)	280 (38.8)	**0.002**
		Agree	53 (35.3)	322 (44.7)	
		Disagree	11 (7.3)	103 (14.3)	
		Strongly Disagree	4 (2.7)	16 (2.2)	
	At times I think I am no good at all.	Strongly Agree	26 (17.3)	66 (9.2)	**<0.001**
		Agree	26 (17.3)	237 (32.9)	
		Disagree	46 (30.7)	241 (33.4)	
		Strongly Disagree	52 (34.7)	177 (24.5)	
	I feel that I have a number of good qualities.	Strongly Agree	91 (60.7)	435 (60.3)	**0.044**
		Agree	47 (31.3)	251 (34.8)	
		Disagree	6 (4.0)	28 (3.9)	
		Strongly Disagree	6 (4.0)	7 (1.0)	
	I am able to do things as well as most other people.	Strongly Agree	78 (52.0)	330 (45.8)	**0.076**
		Agree	59 (39.3)	322 (44.7)	
		Disagree	7 (4.7)	57 (7.9)	
		Strongly Disagree	6 (4.0)	12 (1.7)	
	I feel I do not have much to be proud of.	Strongly Agree	22 (14.7)	73 (10.1)	0.050
		Agree	19 (12.7)	132 (18.3)	
		Disagree	49 (32.7)	280 (38.8)	
		Strongly Disagree	60 (40.0)	236 (32.7)	
	I certainly feel useless at times.	Strongly Agree	23 (15.3)	62 (8.6)	**0.001**
		Agree	20 (13.3)	184 (25.5)	
		Disagree	49 (32.7)	245 (34.0)	
		Strongly Disagree	58 (38.7)	230 (31.9)	
	I feel that I am a person of worth, at least on an equal plane with others.	Strongly Agree	73 (48.7)	273 (37.9)	**0.006**
		Agree	53 (35.3)	340 (47.2)	
		Disagree	11 (7.3)	75 (10.4)	
		Strongly Disagree	13 (8.7)	33 (4.6)	
	I wish I could have more respect for myself.	Strongly Agree	70 (46.7)	347 (48.1)	0.393
		Agree	48 (32.0)	246 (34.1)	
		Disagree	12 (8.0)	66 (9.2)	
		Strongly Disagree	20 (13.3)	62 (8.6)	
	All in all, I am inclined to feel that I am a failure.	Strongly Agree	17 (11.3)	37 (5.1)	**0.023**
		Agree	14 (9.3)	97 (13.5)	
		Disagree	51 (34.0)	263 (36.5)	
		Strongly Disagree	68 (45.3)	324 (44.9)	
	I take a positive attitude toward myself.	Strongly Agree	95 (63.3)	353 (49.0)	**0.010**
		Agree	43 (28.7)	277 (38.4)	
		Disagree	8 (5.3)	73 (10.1)	
		Strongly Disagree	4 (2.7)	18 (2.5)	
UCLA Loneliness Scale	How often do you feel that you lack companionship?	Hardly Ever	77 (51.3)	281 (39.0)	**0.006**
		Some of the Time	54 (36.0)	280 (38.8)	
		Often	19 (12.7)	160 (22.2)	
	How often do you feel left out?	Hardly Ever	80 (53.3)	306 (42.4)	**0.025**
		Some of the Time	50 (33.3)	267 (37.0)	
		Often	20 (13.3)	148 (20.5)	
	How often do you feel isolated from others?	Hardly Ever	70 (46.7)	243 (33.7)	**0.002**
		Some of the Time	51 (34.0)	251 (34.8)	
		Often	29 (19.3)	227 (31.5)	

**Table 5:** Comparisons of Health-related Quality (EQ-5D-5L) Scale for participants with and without any skin diseases (n = 871).

Dimensions and Items		Any Skin Disease				*p*-Value
		No		Yes		
		N	%	N	%	
Mobility	I have no problems in walking about	116	(77.3)	510	(70.7)	0.517
	I have slight problems in walking about	22	(14.7)	129	(17.9)	
	I have moderate problems in walking about	9	(6.0)	68	(9.4)	
	I have severe problems in walking about	1	(0.7)	6	(0.8)	
	I am unable to walk about	2	(1.3)	8	(1.1)	
Selfcare	I have no problems washing or dressing myself	141	(94.0)	665	(92.2)	0.339
	I have slight problems washing or dressing myself	3	(2.0)	31	(4.3)	
	I have moderate problems washing or dressing myself	2	(1.3)	16	(2.2)	
	I have severe problems washing or dressing myself	1	(0.7)	4	(0.6)	
	I am unable to wash or dress myself	3	(2.0)	5	(0.7)	
Activity	I have no problems doing my usual activities	124	(82.7)	456	(63.2)	**<0.001**
	I have slight problems doing my usual activities	16	(10.7)	171	(23.7)	
	I have moderate problems doing my usual activities	8	(5.3)	66	(9.2)	
	I have severe problems doing my usual activities	0	(0.0)	13	(1.8)	
	I am unable to do my usual activities	2	(1.3)	15	(2.1)	
Pain	I have no pain or discomfort	100	(66.7)	319	(44.2)	**<0.001**
	I have slight pain or discomfort	37	(24.7)	257	(35.6)	
	I have moderate pain or discomfort	8	(5.3)	99	(13.7)	
	I have severe pain or discomfort	2	(1.3)	28	(3.9)	
	I have extreme pain or discomfort	3	(2.0)	18	(2.5)	
Anxiety	I am not anxious or depressed	96	(64.0)	309	(42.9)	**<0.001**
	I am slightly anxious or depressed	35	(23.3)	217	(30.1)	
	I am moderately anxious or depressed	12	(8.0)	129	(17.9)	
	I am severely anxious or depressed	3	(2.0)	38	(5.3)	
	I am extremely anxious or depressed	4	(2.7)	28	(3.9)	

**Table 6:** Multiple Linear Regression Models for the factors associated with quality of life based on EQ-5D-5L and mental health (PHQ-9).

Variables	EQ-5D-5L			PHQ-9		
	Coef	SE Coef	*p*-Value	Coef	SE Coef	*p*-Value
Age (years)	−0.0034	0.000910	**<0.001**	−0.1084	0.0214	**<0.001**
Loneliness total score	−0.0331	0.00382	**<0.001**	1.2940	0.0901	**<0.001**
Self Esteem total score	0.0135	0.00154	**<0.001**	−0.3379	0.0363	**<0.001**
Any skin condition (Ref: no)	−0.048	0.0176	**0.006**	1.819	0.414	**<0.001**
Gender (Ref: Men)	−0.0088	0.0145	0.542	1.690	0.340	**<0.001**
Elementary (Ref)						
Intermediate/Secondary	0.1653	0.0597	**0.006**	−1.75	1.41	0.213
University degree	0.1666	0.0587	**0.005**	−2.80	1.38	**0.043**
Postgraduate Degree	0.1404	0.0662	**0.034**	−2.91	1.56	0.062
Single (Ref)						
Married	0.0316	0.0173	0.069	−0.338	0.408	0.408
Divorced/widowed	−0.0563	0.0398	0.157	2.302	0.937	**0.014**
Not Employment (Ref: Employed)	−0.0029	0.0166	0.859	−1.474	0.391	**<0.001**
Any chronic condition (Ref: no chronic conditions)	0.0269	0.0173	0.121	−0.475	0.409	0.245

## Data Availability

This study has no additional supporting data to share.
